# Trajectories of resilience among widows: a latent transition model

**DOI:** 10.1080/13607863.2019.1647129

**Published:** 2019-08-09

**Authors:** Kate Mary Bennett, Davide Morselli, Stefanie Spahni, Pasqualina Perrig-Chiello

**Affiliations:** a School of Psychology, University of Liverpool, UK; b Institute of Social Sciences, University of Lausanne, Switzerland; c Institute of Psychology, University of Berne, Switzerland

**Keywords:** Later life, widowhood, resilience, latent transition analysis

## Abstract

**Objectives:**

In 2015 we identified three profiles of adaptation following spousal bereavement: Vulnerables; Copers and Resilients (Spahni, Morselli, Perrig-Chiello, & Bennett, 2015). However, adaptation to spousal bereavement is a dynamic process. Thus, we examine the trajectories of the same participants longitudinally over two years. We identify the stability and change in profiles of adaptation to widowhood; probability of stability and change; factors that influence trajectories in profile membership.

**Methods:**

Data stem from a longitudinal questionnaire study of 309 older widowed people. The questionnaire included five measures of well-being, serving as the dependent variables of this analysis, and measures of personal resources and contextual factors, including social support, marital happiness, psychological resilience, and demography. Data was analysed using latent transition analysis of the variables loneliness, hopelessness, depressive symptoms, life satisfaction, and subjective health.

**Results:**

The analysis replicated the three Wave 1 profiles as the best theoretical fit: Vulnerables; Copers; and Resilients. Stability was most common, but some participants moved to more or less adaptive profiles, the former being more frequent. Younger age, longer time since widowhood, new life perspectives facilitated adaptation. Those transitioning to less adaptive profiles were more likely to be women and older.

**Discussion:**

The path to adaptation was not linear. Many of the explanatory variables contributed both to positive and negative adaptation. These include previous caring experience, education, psychological resilience and personal strength. This suggests these explanatory variables do not act in isolation but are likely to interact with each other, and with other, yet not measured, factors.

Recent work on older people indicates that many, at least 40%, adapt well to widowhood, either with no noticeable changes in wellbeing before and after bereavement (Bonanno, Wortman, & Nesse, 2004), or returning to pre-bereavement levels of wellbeing relatively quickly (Moore & Stratton, 2003). These widow(er)s have been termed resilient. Researchers have examined resilience in widowed people from three perspectives. First, resilience has been identified as an important psychological trait that promotes wellbeing (Rossi, Bisconti, & Bergman, 2007). Second, Bonanno has argued that resilience represents stability in wellbeing despite bereavement (Bonanno, Wortman, & Nesse, 2004; Galatzer-Levy & Bonanno, 2012). Third, resilience has been defined as the capacity for adaptation and ‘bouncing back’ in the face of adversity (Fuller-Iglesias, Sellars, & Antonucci, 2008; Windle, 2011). These three approaches are brought together in our work, both previously (Spahni et al., 2015) and in the current paper. Underpinning our work is the following definition derived from a large-scale concept analysis of more than 270 papers (Windle, 2011):

Resilience is the process of negotiating, managing and adapting to significant sources of stress or trauma. Assets and resources within the individual, their life and environment facilitate this capacity for adaptation and ‘bouncing back’ in the face of adversity. Across the life course, the experience of resilience will vary (p. 163).

Although many widowed people may demonstrate psychological resilience, research also identifies other outcomes. For example, Bonanno et al. (2004) identified four other outcomes at two-years post bereavement, using depressive symptomatology as the dependent variable: chronic grief; depressed-improved; chronic depression; and common grief. In the same study, at 48 months, all but one of the outcome types remained - common grief disappeared (Galatzer-Levy and Bonanno, 2012). In our earlier cross-sectional work, we used exploratory latent profile analysis across five outcomes: depressive symptoms, loneliness, life satisfaction, hopelessness and subjective health. We identified three groups of widowed participants: Vulnerables (7%); Copers (39%); and Resilients (54%) (Spahni et al., 2015). The profiles of the Vulnerables and Copers were similar but differed by degree, in contrast to the profile of the Resilients. The Copers and Vulnerables had significantly higher levels of depression, loneliness and hopelessness than controls, with the Vulnerables having the highest levels.

A range of factors contribute to successful adaptation, including social support, marital quality, circumstances of death (see for example, Bisconti, Bergeman, & Boker, 2006; Carr et al., 2000; Richardson, 2010). Studies have also shown that even within successfully adaptation depression can increase in situations of financial strain and poor health (Galatzer-Levy & Bonanno, 2012). In our earlier work we found that the resilient widow(er)s were more likely to report higher levels of extraversion, conscientiousness, psychological resilience, agreeableness and lower levels of neuroticism. They also reported higher levels of spousal social support, and higher levels of positive emotional valence. These factors can be understood as resources promoting (or hindering) resilience and contribute to two levels of the ecological model of resilience (individual and community) proposed by Windle and Bennett (2011: see Figure 1). The model has three levels of resources: Individual; Community; and Societal. Individual factors include psychological (such as trait resilience, personality, life philosophy), biological, financial and gender. Community factors include social support, family, social participation. Societal factors include health and welfare services, social policy and culture.

**Figure 1.**
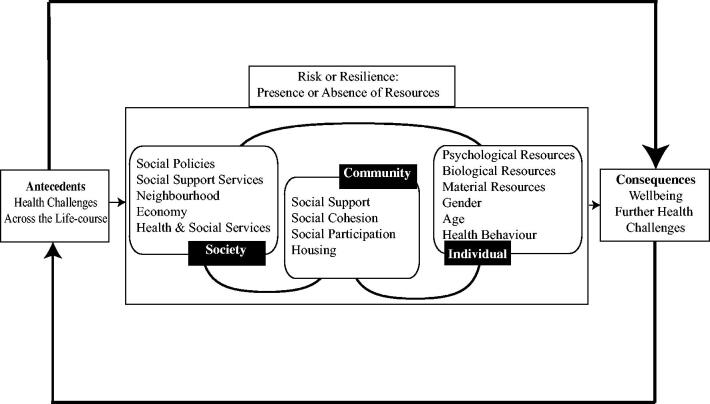
The Ecological Model of Resilience as Applied to Bereavement Adapted from Windle and Bennett (2011).

**Figure 2.**
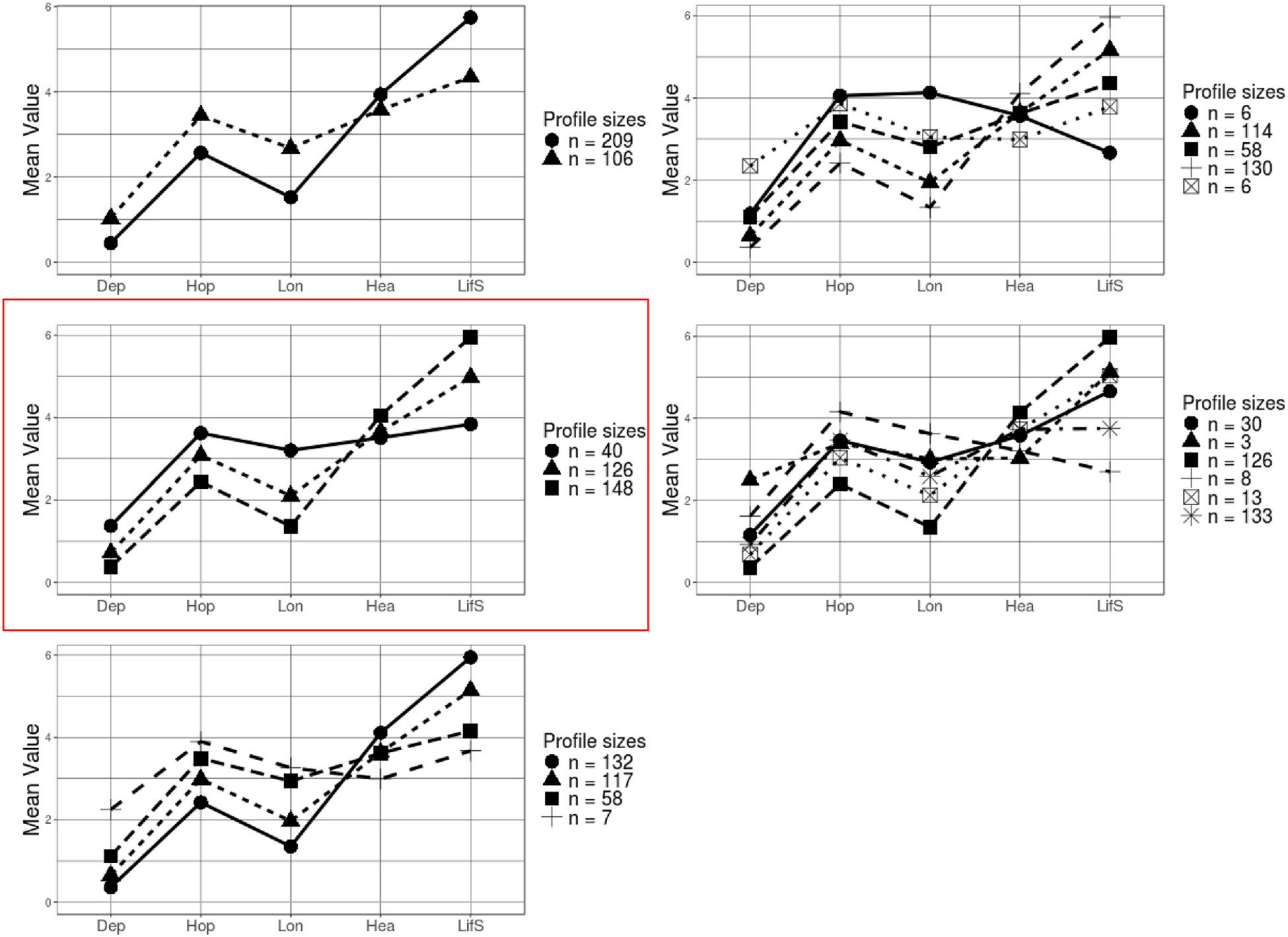
Uncentred means and estimated sizes of 2 to 6 latent profile models of widowed respondents. X-axis Legend: Dep = Depression; Hop = Hopelessness; Lon = Loneliness; Hea = Subjective Health; and LiFS = Life Satisfaction. The 3-profile solution is the focus of subsequent analyses, outlined in the red box. Profiles: ●=Vulnerables; ▲= Copers; ■= Resilients. Resilients have lower levels of Depression, Hopelessness and Loneliness and higher levels of Subjective Health and Life Satisfaction than either the Copers or Vulnerables. The Vulnerables have the highest levels of Depression, Hopelessness and Loneliness and lowest levels of Subjective Health and Life Satisfaction.

Although previous work has identified different trajectories of adaptation to bereavement at a group level, research has not examined trajectories at an individual level. In the current study we examine whether the profiles we identified in our earlier work remain stable between Waves 1 and 2. Second, we examine the probability of participants remaining within a profile, or transitioning to a different one. Third, we examine the factors that influence stability or change in profile membership, and consider it within the ecological model of resilience.

## Method

### Data

The data for this study come from the project ‘Vulnerability and growth: developmental dynamics and differential effects of the loss of an intimate partner in the second half of life’, a longitudinal study exploring the trajectories of psychological adaptation to marital breakup or loss in the second half of life. We report analyses of baseline data (collected in 2012) on the well-being and resources of bereaved individuals who lost their partner within the previous 0–5 years and of the second data wave, two years after (2014). Participants were eligible if they were between 60 and 89 years of age. Respondents were recruited through a random selection from the central register stratified by age group, gender, and marital status, maintained by the Swiss Federal Office of Statistics. Of the 1365 letters of invitation and questionnaires sent to potential participants, 32% agreed to participate in the study. Non-respondents were re-contacted twice. An additional 119 same-aged widowed respondents (94 women, 25 men) were recruited through advertisements and appeals in different media, yielding a sample size of 537 bereaved individuals.

### Sample

Among the widowed sample at Wave 1 402 individuals (228 women; 174 men) fulfilled the criteria of having been married long-term (for fifteen years or more) and being widowed for a maximum of five years. On average, at baseline (Wave 1) the bereaved individuals were 74.41 (*SD* = 7.22) years old, had been married for 45.02 years (*SD* = 9.43) and had lost their partner 3.30 (*SD* = 1.32) years ago. The majority were Swiss (86%, 13% other European, 1% other), and had completed secondary (58%), tertiary (28%) or primary level (14%) education.

The analyses presented here extend the results from an exploratory latent profile analysis (LPA) performed at Wave 1. Two years later these individuals were re-contacted and the same baseline questionnaire was used. Only 1 participant dropped out at Wave 2.

### Measures

The questionnaire included five primary measures of well-being, which served as the dependent variables, and several measures of personal resources and contextual factors. We report Cronbach’s alphas from Wave 1.

#### Dependent variables

*Depression* was measured using an abbreviated version of the Center of Epidemiologic Studies Depression Scale (CES-D) (Hautzinger & Bailer, 1993; Radloff, 1977). Respondents indicated how frequently in the past week they had experienced fifteen depressive symptoms on a four-point scale (0 = ‘not at all’ to 3 = ‘all the time’; α = .86). *Hopelessness* was assessed using a short version of the Hopelessness scales (Beck, Weissman, Lester, & Trexler, 1974; Krampen, 1994), which measures negative expectations of people concerning themselves, their environment and their future. Ten items are rated on a six-point scale (1 = ‘very much untrue’ to 6 = ‘very much correct’; α = .78). *Loneliness* was measured using the short version of the De Jong Gierveld Loneliness Scale (De Jong Gierveld & Van Tilburg, 1999; De Jong Gierveld & Kamphuis, 1985). It consists of six items rated on a five-point scale (1 = ‘no’ to 5 = ‘yes’; α = .86). *Life satisfaction* was assessed with the Satisfaction with Life Scale (Diener, Emmons, Larsen, & Griffin, 1985; Schumacher, 2003). It comprises five items rated on a seven-point scale (1 = ‘completely disagree’ to 7 = ‘completely agree’) and loading onto one factor (α = .87). *Subjective health* was assessed with the widely used question ‘How is your present health?’. The answer options range from 1 = ‘very bad’ to 5 = ‘very good’.

#### Independent variables

*Psychological resilience* was measured with the shortened form of the Resilience Scale (RS-11) (Schumacher, Leppert, Gunzelmann, Straus, & Brähler, 2005; Wagnild & Young, 1993). Eleven items were scored on a seven-point scale (1 = ‘I don't agree’ to 7 = ‘I agree completely’; α = .87), measuring resilience as a unidimensional personal resource. *Personal Growth* was assessed with the short form of the posttraumatic growth inventory (PTGI), consisting of 10 items rated on a six-point scale (1 = ‘I did not experience this chance’ to 6 = ‘I experienced this change to a great degree’) (Cann et al., 2010; Maercker & Langner, 2001). Results of confirmatory factor analysis supported a three-factor solution with a reasonable fit in the current sample (χ^2^ (31) = 176.99, *p* < 0.001, CFI = 0.98, TLI = 0.97, RMSEA = 0.11, WRMR = 1.08), indicating a first factor “*new life perspectives”* (α = .81), a second factor “*spiritual growth”* (α = .91), and a third factor “*personal strength”* (α = .77). *Time since loss* was calculated with the difference between date of loss and the date of participation (in years). *Expectedness* of loss was indicated either as ‘sudden’ = 0 or ‘foreseeable’ = 1. Respondents indicated whether or not the partner was in *need of care previous to death* measured on a 4-point scale (1 = ‘mostly independent’ to 4 = ‘very dependent’).

*Emotional valence of loss* was asked with the question: “The loss of a partner is usually a very painful event. However, circumstances vary greatly from person to person and the loss may be experienced in various ways. How have you personally experienced this loss?” and was answered on a scale from 1 = ‘very negative’ to 10 = ‘very positive’.

In addition, we collected data on personality not utilised in this paper for reasons of space (Big Five Inventory (BFI-10): Rammstedt & John, 2007), and data on *social support*, *length of marriage*, *marital happiness* which we have not analysed as they were non-significant explanatory variables with respect to profile membership at Wave 1.

The analyses controlled for respondents' *age* (in years)*, gender* (0 = male, 1 = female) and *level of education* (from 1 = ‘Primary school’, to 6 = ‘University level’).

For all continuous measures a higher score corresponds to a stronger manifestation.

### Analytic strategy

Following the procedure previously used by Knöpfli, Morselli, and Perrig-Chiello (2016) we tested the hypotheses by means of a latent transition analysis (LTA) in three steps. First, Latent Profile Analysis models were separately performed for each wave on the five outcome variables. Three indicators were used to assess the adequate number of profiles: the sample-size adjusted Bayesian Information Criterion (BIC), the bootstrapped likelihood ratio test (BLRT; McLachlan & Peel, 2000), and entropy. The BIC assumes that a model is penalized by the number of estimated parameters, thus lowest the BIC is the best the model fit. The BLRT indicates whether including one extra class in the analysis produced a significant (i.e., larger than zero) improvement in the model fit. The best fit is indicated by the last significant BLRT coefficient. Entropy indicates the respondents' probability of being classified into more than one profile; values close to 1 indicate high certainty of classification.

In the second step we tested the best fitting transition model, as indicated by Nylund, Asparouhov, and Muthen (2007). Model 1 estimated unconstrained transitions between the profiles at Wave 1 and Wave 2, allowing profiles to having different scores on the outcome variables at the two time points. Model 2 tested instead the measurement invariance of the two sets of profiles. In this model the item-response means of the outcome variables were constrained to be equal across waves. A better fit of Model 2 compared to Model 1 means that the profiles had same configuration and the same interpretation at both time points. Model 3 tested the hypothesis that the profiles represented consecutive stages of psychological adaptation. In this model the probability of backwards transitions was constrained to zero. Members of a profile were allowed only to move upwards to one of the more adapted profile. For instance, respondents of a maladapted profile at Wave 1 could either remain in the same profile or transit only to a more resilient group at Wave 2.

To account for sample attrition and to test whether respondents who dropped out from the survey belonged to a particular profile at Wave 1, we inserted an extra profile at Wave 2 that included dropout respondents. The inclusion of such a profile has the advantage to allowing the use of the full same sample size, assuring that the latent profiles at Wave 1 were the same extracted in previous studies (Spahni et al., 2015). BIC index and entropy were used to estimate the best fitting model.

In the last step of the analysis, distal variables were introduced to the best fitting model to explore the difference among respondents who remained in the same profile and those who transitioned to another profile. This method is similar to the use of distal variables proposed by Lanza, Tan, and Bray (2013) for cross-sectional latent class analysis (LCA) and has been previously applied to latent transition models by Knöpfli et al. (2016). The model was estimated following Nylund-Gibson, Grimm, Quirk, and Furlong (2014) and Asparouhov and Muthén (2013) recommendations. The profile probabilities of the most likely profile membership for each wave was used to calculate the classification uncertainty rate (CUR) at the two time points. The CUR is the average probability that members of each class could be classified also in the other classes, and was computed as the logarithm of the proportion between the average probability of the most likely profile and the sum of the average probabilities of the other profiles (Asparouhov & Muthén, 2013). The CUR was then used to correct the classification of respondents into the profiles and to estimate the transitions from each profile at Wave 1 to each profile at Wave 2. Finally, distal variables were inserted in the model one at the time and their means was estimated for each possible transition pattern. This procedure allowed us to correct the respondents’ classification by CUR at each wave, and had the advantage of accounting for the classification error at both time points, contrary to models that analyse the transitions between the most likely profiles Wave 1 and Wave 2.

Analyses were performed with Mplus 7.3 (Muthén & Muthén, 1998-2010) in combination with the MplusAutomation package for R (Hallquist & Wiley, 2014). Models were estimated with maximum likelihood estimation with standard errors based on the first-order derivatives, as implemented in Mplus. To ensure that model results did not depend on local maxima, the each final model was reproduced by increasing to 1000 the number of random starts and to 100 the number of final-stage optimizations (Hipp & Bauer, 2006; Marsh, Luedtke, Trautwein, & Morin, 2009).

## Results

### Latent profiles of responses to bereavement and longitudinal transitions

First, the two waves were analysed separately to estimate the best number of profiles at each time point. At Wave 1, the results replicated Spahni et al. (2015) identifying 3-profiles. With respect to Wave 2, the BIC and BLRT indicators suggested that the best fit of the data was provided instead by 5 profiles (Table 1). Two resilient groups with fairly similar scores were found, and the vulnerable respondents were split into two groups, one of which could be described as at high risk (i.e., high levels of loneliness and hopelessness, and low levels of life satisfaction, Figure 2). However, the small size of this group (*n* = 5) and the similarities between the two resilient profiles convinced us that the best model for our analyses was once again the 3-profiles model. This model had the highest entropy, suggesting a small classification error, and thus a higher reliability of the analyses of transitions between profiles.

**Table 1.** Fit indices for tested Time 2 models

N of Profiles	BIC	BLRT	Entropy
2	2845.93	−1561.502***	.84
3	2774.14	−1362.562***	.87
4	2729.31	−1309.413***	.78
5	2721.77	−1269.74***	.81
6	2734.26	−1248.711*	.79

^*^ *p* < .05.

^***^ *p* < .001.

After determining the appropriate number of profiles for both waves we tested three latent transition models as described in the previous section. Results are reported in Table 2. Profiles were sorted from the most vulnerable to the most resilient to facilitate interpretation. In Model 1 (BIC = 15631.40, Entropy = .82) all the outcome variables were unconstrained between the two waves. Dropout respondents at Wave 2 represented only a marginal percentage of the sample, and for this reason they were excluded in subsequent analyses.

**Table 2.** Transition probabilities of Wave 1 profiles (Rows) by Wave 2 Profiles (Columns)

Profiles		Vulnerables	Copers	Resilients	Dropouts
Model 1		*n* = 50	*n* = 151	*n* = 200	*n* = 1
Vulnerables	*n* = 33	0.791	.174	.034	.000
Copers	*n* = 161	.185	.711	.103	.000
Resilients	*n* = 208	.014	.059	.922	.005
Model 2		*n* = 33	*n* = 155	*n* = 213	*n* = 1
Vulnerables	*n* = 34	.680	.281	.039	.000
Copers	*n* = 158	.106	.744	.150	.000
Resilients	*n* = 210	.002	.060	.933	.005
Model 3		*n* = 29	*n* = 160	*n* = 212	*n* = 1
Vulnerables	*n* = 36	.668	.273	.059	.000
Copers	*n* = 159	.000	.866	.134	.000
Resilients	*n* = 207	.000	.000	.995	.005

*Note:* Profile counts are estimated on their most likely latent profile pattern; Except for the dropout profile which is observed, all other categories are estimated a latent variable and thus the individual probability to belong to each profile can slightly change from model to model.

In Model 2, the outcome variables were constrained to be equal across waves for each profile. The fit of Model 2 (BIC = 15565.01, Entropy = .83) suggested that the profiles had the same structure and same interpretation across the two time points. In Model 3 we tested whether respondents moved primarily from maladapted profiles to more resilient ones, without allowing backward transitions. To test this hypothesis, Model 3 constrained to zero all backward transitions. Model 3 fit the data (BIC = 15580.40, Entropy = .88) more poorly than Model 2, suggesting that bereaved respondents moved from lower adapted statuses to higher adapted, but also vice-versa. Model 2 was retained for further analyses on distal variables, in order to explore difference among respondents who did not change profile over the two observations, those who moved upward, and those who moved backward.

### Differences among transition patterns

Differences among transition patterns were explored following the procedure described in the analytical strategy section. Given the relatively small number of observations some transition cells were either empty or with a small number of cases. For this reason we used a non-parametric analytical strategy, by computing Cohen (1988) *d* to evaluate the size of the difference between respondents who were classified in the same profile at both time points and those who moved to higher or lower profiles. Cohen's *d* was also used to compare differences between respondents classified twice as vulnerable and twice as resilient. Results are reported in Table 3; transitions with only one or two respondents are reported but not commented on.

**Table 3.** Means of socio-demographic, context, and inter-individual variables for each transition pattern

		Vulner *W2*	Copers *W2*	Resili *W2*
Variable	Profile	*M* (*SE*, *n*)	*M* (*SE*, *n*)	*M* (*SE*, *n*)
Age	Vulner*W1*	74.49 (1.78, 23)	70.63 (7.6, 4)	68.72 (9.68, 2)
	Copers*W1*	72.48 (3.11, 15)	76.26 (0.78, 121)	72.32 (4.48, 5)
	Resili*W1*	82.09 (12.55, 3)	77.51 (4.8, 16)	73.38 (0.6, 212)
Men	Vulner*W1*	0.42 (0.18, 20)	0.34 (0.48, 7)	0.46 (0.64, 2)
	Copers*W1*	0.41 (0.22, 18)	0.45 (0.1, 112)	0.83 (0.74, 19)
	Resili*W1*		0.15 (0.75, 21)	0.41 (0.15, 202)
Level of education	Vulner*W1*	3.2 (0.37, 22)	2.66 (1.25, 5)	5.41 (2.37, 2)
	Copers*W1*	3.52 (0.67, 17)	3.71 (0.15, 112)	2.09 (0.97, 7)
	Resili*W1*	5.59 (9.67, 1)	2.78 (0.7, 18)	3.79 (0.1, 217)
Unexpectedness	Vulner*W1*	0.29 (8.04, 18)	0.87 (14.13, 9)	0.97 (22.08, 2)
	Copers*W1*	0.28 (8.55, 18)	0.34 (5.98, 112)	0.57 (2.9, 14)
	Resili*W1*		0.57 (2.81, 12)	0.37 (4.85, 216)
Months since loss	Vulner*W1*	37.13 (3.53, 23)	48.93 (16.25, 3)	17.47 (15.65, 3)
	Copers*W1*	31.04 (5.17, 18)	39.85 (1.67, 112)	17.82 (15.08, 10)
	Resili*W1*		33.25 (8.28, 7)	42.03 (1.27, 225)
Dependency of the spouse	Vulner*W1*	3.6 (0.67, 22)	1.35 (2.24, 5)	1.39 (67.59, 2)
	Copers*W1*	2.72 (0.35, 18)	3.22 (0.19, 118)	2.79 (0.81, 5)
	Resili*W 1*		3.25 (0.82, 20)	2.89 (0.12, 211)
Psychological Resilience*W2*	Vulner*W1*	3.95 (0.14, 25)	6.09 (0.59, 3)	4.68 (1.07, 2)
	Copers*W1*	5.73 (0.33, 13)	5.1 (0.08, 115)	5.13 (0.41, 19)
	Resili*W1*	6.17 (3.99, 3)	4.57 (0.37, 17)	5.89 (0.07, 204)
New Life Perspectives*W2*	Vulner*W1*	3.93 (0.43, 20)	4.41 (1.84, 5)	1.98 (2.26, 4)
	Copers*W1*	3.09 (0.46, 16)	3.42 (0.17, 120)	4.26 (0.94, 10)
	Resili*W1*	5.16 (3.1, 2)		3.72 (0.1, 224)
Spiritual Growth *W 2*	Vulner*W1*	3.11 (0.53, 20)	3.26 (1.75, 7)	2.45 (16.27, 2)
	Copers*W1*	2.38 (0.77, 18)	2.61 (0.23, 112)	3.7 (1.01, 14)
	Resili*W1*		4.61 (1.49, 6)	2.76 (0.15, 222)
Personal Strength *W 2*	Vulner*W1*	3.32 (0.39, 21)	4.58 (1.36, 7)	2.51 (7.29, 1)
	Copers*W 1*	3.4 (0.6, 17)	3.72 (0.18, 122)	4.17 (0.93, 22)
	Resili*W1*	1.62 (13.29, 1)	4.56 (1.08, 13)	3.98 (0.12, 197)

*Note:* Empty cells represent transitions with no respondents; *n is* the estimated most likely classification; Vulner = Vulnerables; Resili = Resilients; *Wave 1* and *Wave 2* refer to Wave 1 and Wave 2, respectively.

With respect to the socio-demographic variables, there were no noticeable age differences among respondents that remained in the same profile between Wave 1 and Wave 2. However, Wave 1 Vulnerables who were classified as Copers at Wave 2 were marginally younger than those who remained in the vulnerable profile (*d* = .41). Similarly, Resilients at Wave 1 who became Copers were marginally older than those who remained resilient (*d* = .42). This pattern was not confirmed by Copers: younger respondents were classified as either vulnerable or resilient at Wave 2 (*d* = .41 and *d* = .46, respectively). Stable respondents were evenly distributed among men and women in all three profiles. On the contrary, Copers moving to Resilients were mainly men, and back-stepping Resilients were mostly women. However, the sizes of the effects were marginal (*d* = .24 and *d* = .10, respectively) and no gender difference can be statistically assumed.

Concerning the level of education, the stable Vulnerables tended to have a lower average education than the stable Resilients (*d* = .39). Respondent who moved, irrespective of the direction of the adaptation profiles, tended to have a lower levels of education than stable respondents. For instance, if a marginal difference was found between stable Vulnerables and Vulnerables moving to Copers (*d* = .28), a large difference was found for Copers moving to Resilients (*d* = 1.00) and vice versa (*d* = .61).

When looking at context of the loss, there was a small difference concerning the time of the loss, with respondents classified twice as Resilients having experienced the loss more remotely than respondents twice classified as vulnerable (*d* = .26). In addition, backward classifications were associated with smaller time distances since the event (*d* = .48 for Wave 1 Copers, and *d* = .47 for Wave 1 Resilients), while more time had passed for vulnerable moving to Copers (*d* = .65). Respondents who moved to the resilient profile had experienced the event on average within the last 18 months (*d* = 1.10 for Wave 1 Vulnerables, *d* = 1.03 for Wave 1 Copers).

Only a small percentage of stable respondents (i.e., about one third of each profile) had an unexpected loss. Although the average unexpectedness for respondents changing profile between Wave 1 and Wave 2 was higher than the stable ones, the Cohen's *d* were very small (*d* < 05) and thus these differences should be interpreted as statistically non reliable. With respect to the conditions of the ex-partner in the last months of life, for stable Resilients the partner needed less care than for stable Vulnerables (*d = .*38). Vulnerables who moved to the coper profile also reported a smaller need of care of the partner before dying (*d* = .68). Marginal differences of dependency were also reported by Copers who either moved forward or backward (*d* = .22, and *d* = .23 respectively), while a higher need for care was reported by Resilients moving backwards, but the effect was very small (*d* = .11).

Concerning intra-individual differences, psychological resilience was lowest for the Vulnerables who remained vulnerable, and highest among the Resilients. The difference between stable vulnerable and stable Resilients was very large (*d* = 2.1). High scores of psychological resilience were also reported by Wave 1 Vulnerables moving to the coper profile at Wave 2 (*d* = 3.03). In contrast, lower scores were reported by Wave 1 Resilients moving to coper (*d* = 1.30). Marginally higher score were found for Resilients moving to Vulnerables (*d* = .24). However, the standard error of psychological resilience for this group was relatively large, and this score should be considered cautiously. In addition, Wave 1 Copers moving to Vulnerables reported higher psychological resilience (*d* = .68).

No notable differences were found in the perception of having developed new life perspectives after the event among the three stable patterns. More accentuated growth was reported by vulnerable moving to Copers (*d* = .20) and Copers moving to Resilients (*d* = .40), but contrary to expectations vulnerable moving to Resilients reported lower scores on this measure (*d* = .80). However, the standard error for this group was relatively large, and this result should be interpreted cautiously. Higher spiritual growth was reported by stable Vulnerables than the stable Resilients, but the size effect of the difference was marginal (*d* = .15). Higher spiritual growth was also reported by Copers passing to Resilients (*d* = .42) and Resilients moving to Copers (*d* = .85). Stable Resilients reported higher growth in personal strength than respondents that remained in the vulnerable profile (*d* = .40). Vulnerable respondent passing to Copers scored higher than stable Vulnerables (*d* = .54), and Copers passing to Resilients scored higher than stable Copers (*d* = .18). Resilient back-stepping to Copers also scored higher than stable Resilients (*d* = .31), while lower growth in personal strength was reported by Copers moving to vulnerable (*d* = .16), although both score differences were small.

## Discussion

We confirmed that the three profile solution identified in our earlier work was the most appropriate. Participants were classified as Resilients, Copers or Vulnerables. Stability was the most probable outcome: those who had been Resilients remained Resilients, Copers remained Copers, and Vulnerables remained Vulnerables. We confirmed that Resilients had lower depression, hopelessness, loneliness, higher life satisfaction and subjective health compared to the other groups. Both Copers and Vulnerables had higher levels of depression, hopelessness, loneliness, and lower life satisfaction and subjective health, with the Vulnerables having more extreme values.

Some participants moved either upwards into a more adaptive class, or downwards into a less adaptive one. The latter being the least probable outcome. In general, where participants did move class it was only to the next class. This finding has not been demonstrated before.

It is not surprising that the majority of participants remain in the same profile over time. This is in line with other findings, especially those of Galatzer-Levy and Bonanno (2012), especially since the largest profile at Wave 1 were Resilients. What is interesting both theoretically and in terms of intervention, are the participants who moved to a more adaptive or to a less adaptive class. What are the factors that facilitate or hinder a move to resilience? Can we understand those factors in the light of the Ecological Model of Resilience?

The results suggest those who move to a more adaptive class are more likely to be younger and are more likely to be men, whilst those moving to a less adaptive class are more likely to be older and women. In terms of intervention neither of these variables are amenable to change. However, they are useful indicators of where resources need to be targeted. Those who adapt are more likely to have positive life perspectives. This is a resource which has been identified as facilitating adaptation and resilience elsewhere and is potentially amenable to change. Indeed, it has been used in the operationalisation of resilience elsewhere (Donnellan, Bennett, & Soulsby, 2015; Moore & Stratton, 2003), and can be seen as an individual level factor in the Ecological Model of Resilience (Windle & Bennett, 2011).

The remaining explanatory variables demonstrate the complexity and non-linearity of adaptation to widowhood. Greater independence from care responsibilities prior to bereavement facilitates positive adaptation from Vulnerable to Coper, and indeed Resilient, it also is associated with negative adaptation in Copers who move to Vulnerable. This may explain why previous research is equivocal about the impact of prior caring (Boerner, Schulz, & Horowitz, 2004; Richardson, 2010). One would predict that psychological resilience would be higher amongst those in the stable Resilient class and would be associated with moving to a more adaptive class, and this is the case. However, higher psychological resilience is also found amongst those participants moving from the Coper to the Vulnerable class. Similarly, high levels of Spiritual Growth are associated both with both more and less adaptive transitions. Finally, lower educational status is more likely both in Vulnerables and in those who moved to both a more adaptive and less adaptive class. These findings suggest that these factors do not influence adaptation in isolation but rather interact with each other, and with other, not yet examined variables. Although, we have not examined these interactions in this study, the results suggest that they may be considered in the light of the Ecological Model of Resilience which argues that factors interact with each other, and that resilience is a dynamic process which is dependent on the presence, absence and access to resources (Donnellan et al., 2015; Ungar, 2011; Windle & Bennett, 2011).

In parallel with the dynamic nature of resilience is the dynamic nature of bereavement per se. One of the most interesting findings of this study concerns the effect of time since loss. Those in the stable resilient class are more likely to have been widowed longer. This suggests resilience not only in bereavement processes, but also in widowhood [a distinction made by Bennett, Hughes, & Smith (2005)]. The evidence suggests that adjustment to widowhood does not follow a linear time course; those moving upwards to the Resilient class (n = 13) have been widowed less time than those moving backwards (n = 20). There may be oscillation in adaptation, and this may reflect the oscillation between loss-focussed and restoration-oriented coping identified in the Dual Process Model of Bereavement (Stroebe & Schut, 1999).

There are some limitations to this work. First, we do not have pre-loss data for our participants. However, at Wave 1 we utilised a married control group, and we now have longitudinal post-loss data for our widowed participants. Second, we use a self-constructed single-item question for dependency, and it is known that caregiving and dependency influence bereavement trajectories (Stroebe & Boerner, 2015). Third, as we have suggested it is likely that the explanatory factors interact with each other, and we have not analysed the data in that way in this paper. This is an area for future research. Fourth, whilst we have been able to consider some community and individual level resources from the Ecological Model of Resilience, some other resources, especially societal level resources have not been available for inclusion. Again, this an area of future research.

To conclude, we demonstrate that patterns of adaptation to widowhood are relatively stable over time. However, some participants continue to adapt positively, whilst others have negative trajectories. Men and younger participants are more likely to adapt positively than women or older participants. The contributions of both psychological factors and prior care responsibilities are mixed, i.e. they influence both positive and negative adaptation. We argue that this is likely because of their complex interplay in the lives of widowed older people. These complexities may be understood in the light of the Ecological Model of Resilience. The study illustrates the importance of following the trajectories of individual widowed people, since the patterns of adjustment are not always linear. The study also points to the importance of targeting specific groups of widowed people for intervention, rather than focussing resources on all older widowed people.
